# Supplementary material for: Current and projected regional economic impacts of heatwaves in Europe
Source: Nat Commun. 2021 Oct 4;12:5807. doi: 10.1038/s41467-021-26050-z (PMC8490455; doi:10.1038/s41467-021-26050-z)
Supplement: Supplementary file 1 — Supplementary Information [file 41467_2021_26050_MOESM1_ESM.pdf]

Supplementary Information

Current and projected regional economic impacts of heatwaves in Europe

David García-León*, Ana Casanueva, Gabriele Standardi, Annkatrin Burgstall, Lars Nybo, Andreas F. Flouris

*Corresponding author: David García-León, email: david.garcia-leon@ec.europa.eu. Postal address: European Commission, Joint Research Centre, Edificio Expo, Inca Garcilaso 3, 41092 Seville, Spain.

Contents

Supplementary Figures (Supplementary Figures 1-5)
Supplementary Tables (Supplementary Tables 1-3)
Supplementary Discussion
Supplementary References

Supplementary Figures

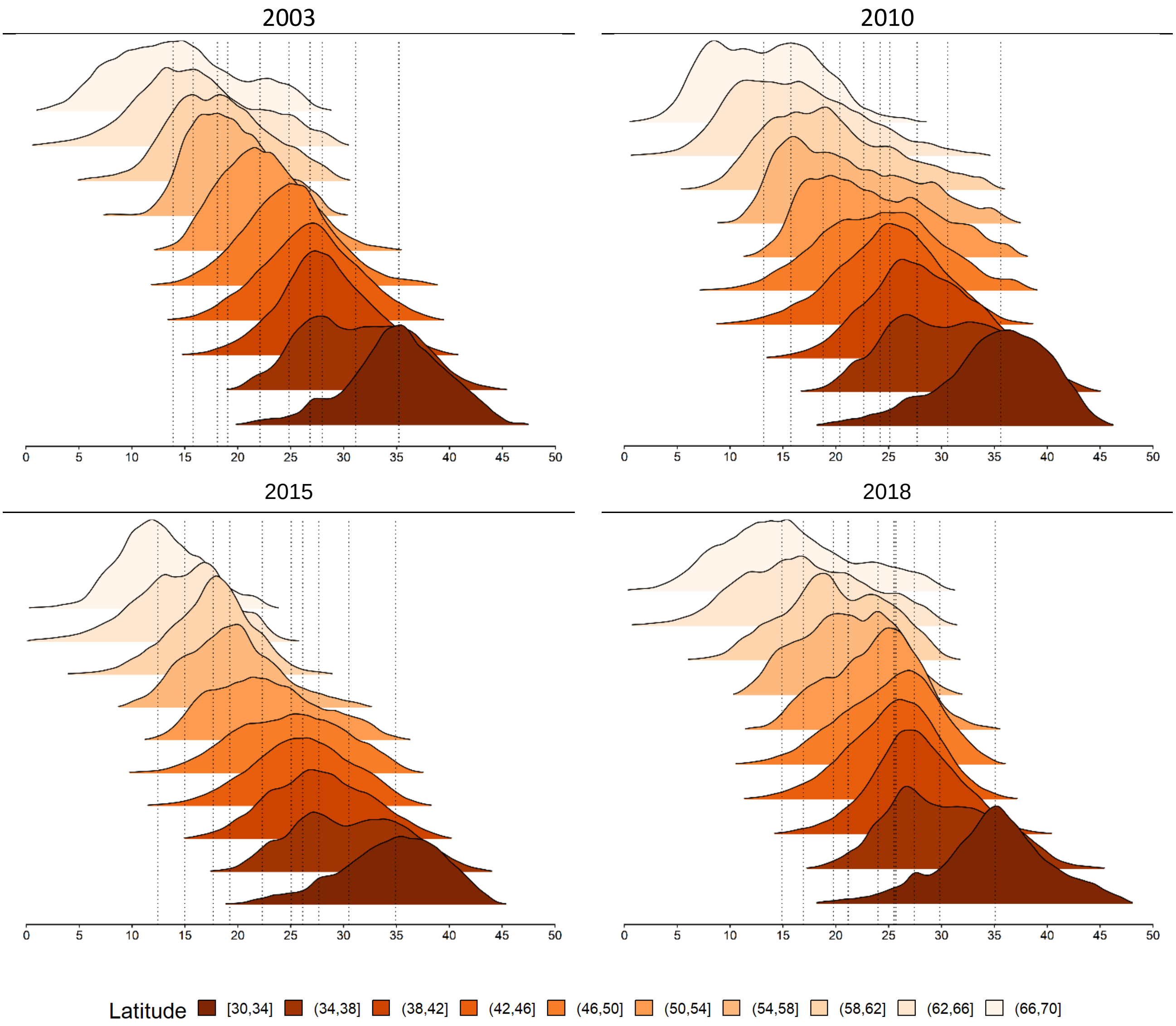

Supplementary Figure 1. Spatial heterogeneity of extreme temperatures. Distribution of maximum temperatures (in ºC) during summer months (JJA) at different latitude ranges in the four years studied. The dotted lines represent the median maximum temperature of each distribution, reflecting the usual North-South gradient in temperatures, but capturing at the same time a certain degree of interannual spatial variability.

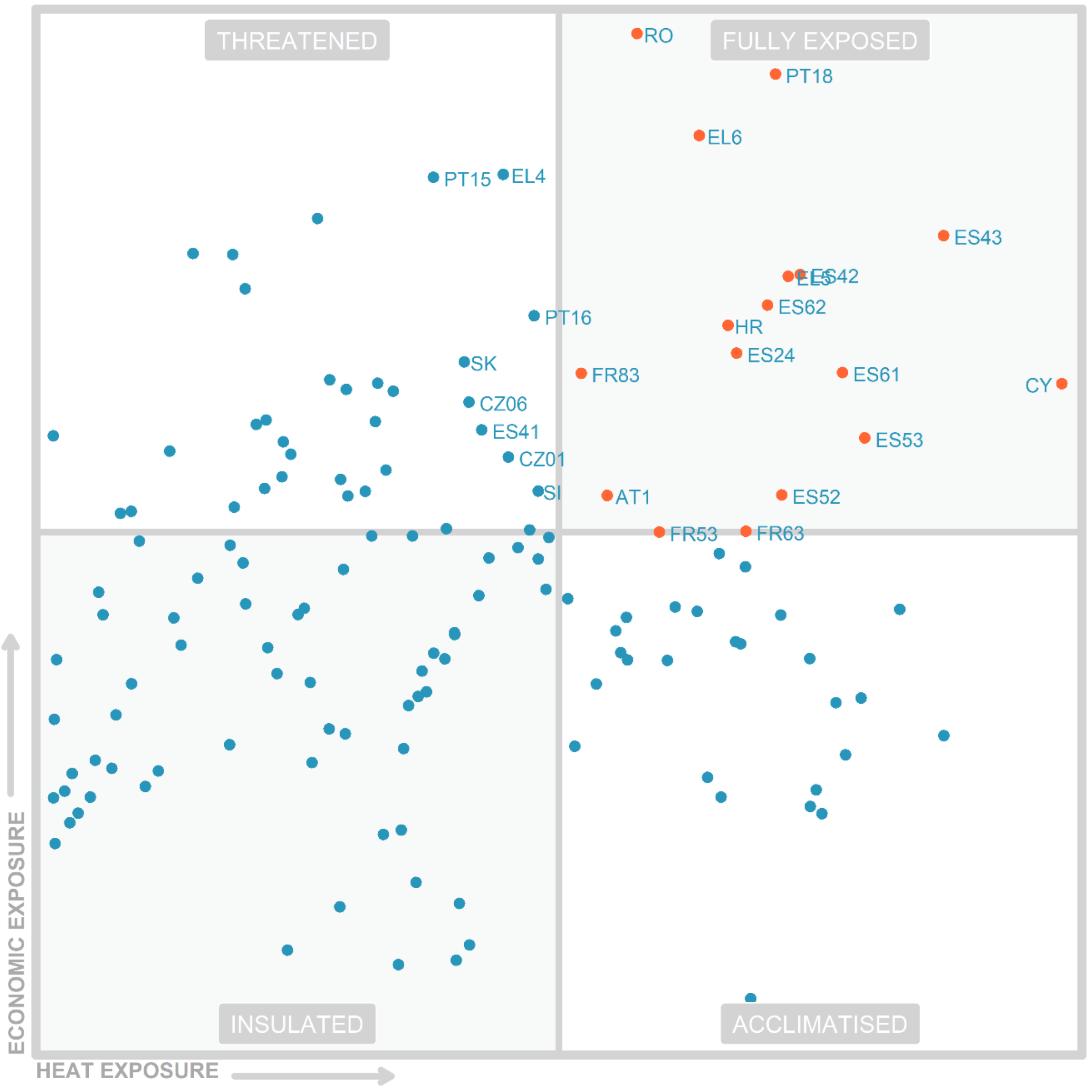

Supplementary Figure 2. Spatial heterogeneity of extreme temperatures. Regional vulnerability to heatwaves. Classification of regions according to their average cumulative heat exposure (average $WBGT_{sun}$ during heatwaves in 2003, 2010, 2015, and 2018) and their economic exposure (=Share of outdoor activities out of total economic activities). Four groups of regions are identified: ‘insulated’, ‘threatened’, ‘acclimatised’ and ‘fully exposed’. ‘Fully exposed’ and ‘threatened’ regions are subject to increasing heat stress risk due to climate change. ‘Insulated’ regions are not expected to suffer frequent heat stress damages in the future, as they show low environmental and economic exposure. Meanwhile, the economies of ‘acclimatised’ regions, despite being highly exposed to heat, are mainly based on indoor activities. We do not foresee regions lying in the lower quadrant transitioning to the upper part. In our view, left-right transitions are much more likely in the medium term due to increasingly warming temperatures. Fully exposed regions (high heat and economic exposure) are highlighted in orange. Refer to Supplementary Table 2 for regions abbreviations.

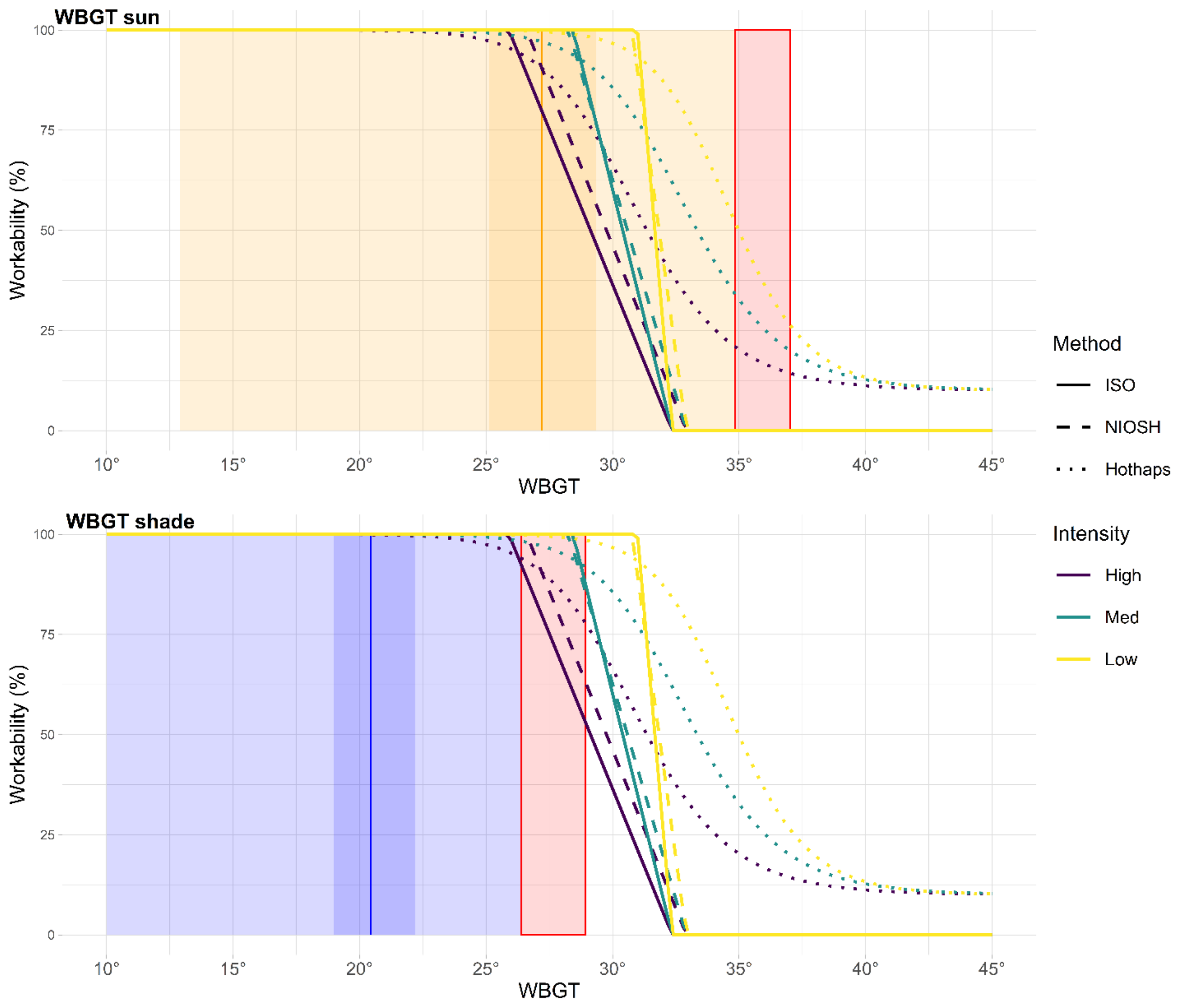

Supplementary Figure 3. **Heat-to-Workability transfer functions.** Comparison of the three approaches considered, illustrating the biophysical link between heat and labour productivity losses under different working load intensities (in Watts). The distribution of the daily average values of WBGT (sun: outdoor; shade: indoor) during identified heatwaves is also depicted. Shaded areas represent the different quartiles (0,25,50,75,100) of the respective WBGT distributions, while the vertical solid lines represent the medians. Red-shaded areas represent the right tail shift in the distributions of WBGT projected by the selected climate models (MPICSC-REMO2 and KNMI-RACMO). This picture illustrates how, under current heat conditions, indoor economic activities are hardly affected by heat-induced productivity losses for any of the heat transfer functions considered. In contrast, over the next decades indoor workers will begin to be affected by productivity losses in response to excessive heat, especially in southern regions.

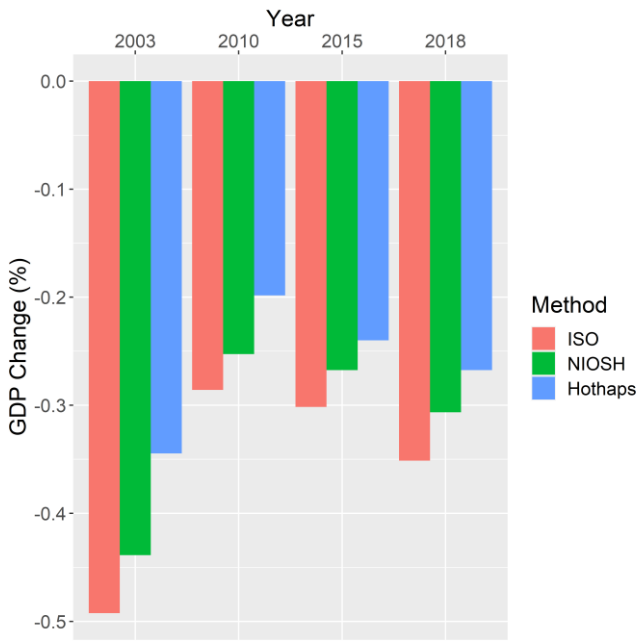

Supplementary Figure 4. GDP losses by heat transfer functions. Aggregated GDP impacts of heatwaves according to the three heat-to-workability functions considered. Differences within the three approaches were proportional and responded to the construction of the respective workability functions, being the Hothaps approach the most conservative out of the three considered.

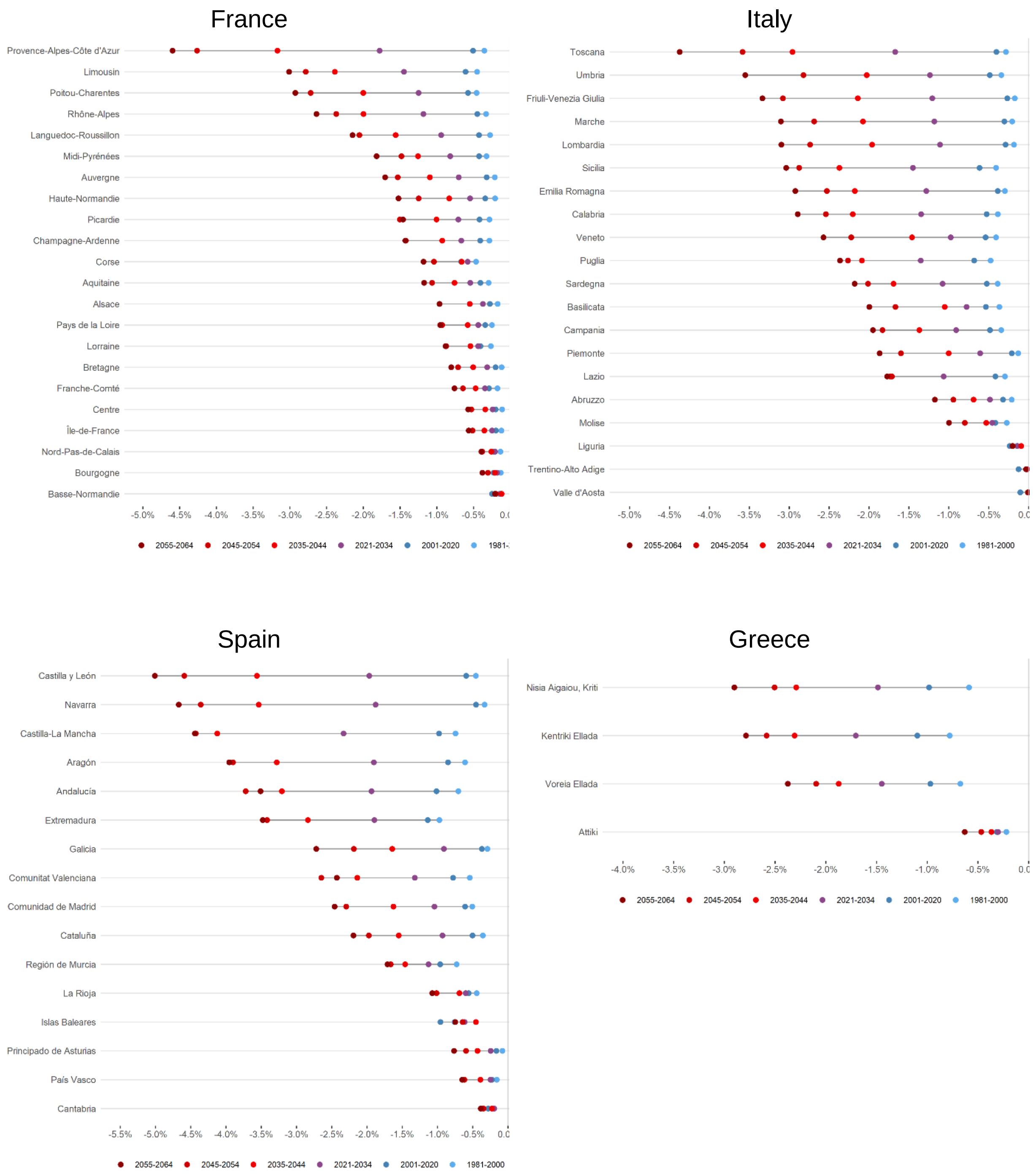

Supplementary Figure 5. **Regional-level projected impacts.** Analogously to Fig. 4, the projected evolution of heatwave-induced economic damages is shown at the regional level in four different southern European countries. The projected trend in damages is positive in all regions, with more exposed (environmentally and economically) regions being projected to experience more acute damages.

Supplementary Tables

Supplementary Table 1. **Heatwave frequency per region and year**. Most affected regions by total number of heatwaves events identified and total cumulative duration (in days). Refer to Supplementary Table 2 for regions abbreviations.

**2003**	Region	Events	Duration
	DE27	7	76
	EL62	3	75
	DE14	8	75
	ITI1	4	74
	ITC3	3	73
	DE13	8	73
	MT00	3	71
	ITH1	6	70
	FRF1	7	70
	ITH3	4	69
	ITC1	5	69
	FRM0	4	69
	ES53	5	69
	ITI4	5	68
	FRL0	4	68
	ITI2	5	67
	ITH4	3	67
	ITG2	5	67
	SI04	7	66
	ITC4	6	66
	DE12	7	66
	ITH2	7	65
	DE71	6	65
	DE21	8	65
	ITI3	7	64

**2010**	Region	Events	Duration
	EL42	2	53
	LV00	4	50
	FI1C	4	50
	EE00	4	50
	FI1B	3	47
	EL41	4	47
	FI19	4	45
	EL51	4	45
	EL65	4	44
	EL52	4	44
	ES61	4	43
	BG33	4	42
	FI20	2	40
	ES43	5	40
	ES30	5	40
	EL30	6	40
	IS	6	39
	EL61	5	39
	BG42	4	39
	RO32	3	38
	RO31	3	38
	RO22	3	38
	LT02	4	38
	FI1D	4	38
	ES42	4	38

**2015**	Region	Events	Duration
	EL62	2	68
	RO21	7	57
	MT00	3	55
	ITF5	3	55
	HR03	6	55
	EL42	3	54
	SK03	6	53
	RO12	6	53
	RO11	6	52
	ITI1	5	52
	EL52	4	52
	ITH1	4	50
	ITC3	5	50
	HU32	6	50
	HU31	6	50
	EL51	3	50
	RO42	6	49
	RO32	5	48
	ITH4	5	48
	ITF6	3	48
	ITF2	5	48
	ES61	3	48
	EL61	5	48
	EL41	4	48
	RO22	5	47

**2018**	Region	Events	Duration
	SE22	5	71
	NL13	9	70
	SE23	4	69
	DK03	8	69
	SE21	6	68
	NL21	7	66
	DK04	7	66
	DEA2	9	66
	SE12	5	65
	PL81	8	65
	FRE2	8	65
	DK01	6	65
	DEA5	8	65
	DEA3	7	65
	DE60	12	65
	BE34	9	63
	FRE1	7	62
	DEA4	9	62
	DE92	9	62
	SE31	6	61
	DK02	5	61
	DE93	10	61
	BE32	7	61
	LU00	7	60
	DEF0	8	60

Supplementary Table 2. **Regions analysed and correspondence with the regional resolution of the economic model**. 274 regions were analysed*. Heatwave characterisation (events, duration, severity) and labour productivity losses were calculated at the regional level. Productivity losses were spatially aggregated (weighted by population) according to the shown spatial resolution of the regions considered in the economic model. *Canary Islands (NUTS2: ES70) were excluded, as they are outside the climate models' domain. The autonomous cities of Ceuta (ES63) and Melilla (ES64) were grouped within the region of Andalusia (ES61).

NO. REGION	COUNTRY CODE	NUTS CODE	NUTS NAME	NUTS LEVEL IN CGE MODEL	CGE CODE
1	AT	AT11	Burgenland	1	AT1
2	AT	AT12	Niederösterreich	1	AT1
3	AT	AT13	Wien	1	AT1
4	AT	AT21	Kärnnten	1	AT2
5	AT	AT22	Steiermark	1	AT2
6	AT	AT31	Oberösterreich	1	AT3
7	AT	AT32	Salzburg	1	AT3
8	AT	AT33	Tirol	1	AT3
9	AT	AT34	Vorarlberg	1	AT3
10	BE	BE23	Prov. Oost-Vlaanderen	1	BE2
11	BE	BE24	Prov. Vlaams-Brabant	1	BE2
12	BE	BE25	Prov. West-Vlaanderen	1	BE2
13	BE	BE31	Prov. Brabant Wallon	1	BE3
14	BE	BE32	Prov. Hainaut	1	BE3
15	BE	BE33	Prov. Liège	1	BE3
16	BE	BE34	Prov. Luxembourg (BE)	1	BE3
17	BE	BE35	Prov. Namur	1	BE3
18	BE	BE10	Région de Bruxelles-Capitale/Brussels Hoofdstedelijk Gewest	1	BE1
19	BE	BE21	Prov. Antwerpen	1	BE2
20	BE	BE22	Prov. Limburg (BE)	1	BE2
21	BG	BG31	Северозападен	0	BG
22	BG	BG32	Северен централен	0	BG
23	BG	BG33	Североизточен	0	BG
24	BG	BG34	Югоизточен	0	BG
25	BG	BG41	Югозападен	0	BG
26	BG	BG42	Южен централен	0	BG
27	CH	CH	Confédération suisse	0	CH
28	CY	CY00	ΚΥΠΡΟΣ	0	CY
29	CZ	CZ07	Střední Morava	2	CZ07
30	CZ	CZ08	Moravskoslezsko	2	CZ08
31	CZ	CZ01	Praha	2	CZ01
32	CZ	CZ02	Střední Čechy	2	CZ02
33	CZ	CZ03	Jihozápad	2	CZ03
34	CZ	CZ04	Severozápad	2	CZ04
35	CZ	CZ05	Severovýchod	2	CZ05
36	CZ	CZ06	Jihovýchod	2	CZ06
37	DE	DE50	Bremen	1	DE5
38	DE	DE60	Hamburg	1	DE6

39	DE	DE71	Darmstadt	1	DE7
40	DE	DE72	Gießen	1	DE7
41	DE	DE73	Kassel	1	DE7
42	DE	DE80	Mecklenburg-Vorpommern	1	DE8
43	DE	DE91	Braunschweig	1	DE9
44	DE	DE92	Hannover	1	DE9
45	DE	DE93	Lüneburg	1	DE9
46	DE	DE94	Weser-Ems	1	DE9
47	DE	DE11	Stuttgart	1	DE1
48	DE	DE12	Karlsruhe	1	DE1
49	DE	DE13	Freiburg	1	DE1
50	DE	DE14	Tübingen	1	DE1
51	DE	DE21	Oberbayern	1	DE2
52	DE	DE22	Niederbayern	1	DE2
53	DE	DE23	Oberpfalz	1	DE2
54	DE	DE24	Oberfranken	1	DE2
55	DE	DE25	Mittelfranken	1	DE2
56	DE	DE26	Unterfranken	1	DE2
57	DE	DE27	Schwaben	1	DE2
58	DE	DE30	Berlin	1	DE3
59	DE	DE40	Brandenburg	1	DE4
60	DE	DEA1	Düsseldorf	1	DEA
61	DE	DEA2	Köln	1	DEA
62	DE	DEA3	Münster	1	DEA
63	DE	DEA4	Detmold	1	DEA
64	DE	DEA5	Arnsberg	1	DEA
65	DE	DEB1	Koblenz	1	DEB
66	DE	DEB2	Trier	1	DEB
67	DE	DEB3	Rheinhessen-Pfalz	1	DEB
68	DE	DEC0	Saarland	1	DEC
69	DE	DED2	Dresden	1	DED
70	DE	DED4	Chemnitz	1	DED
71	DE	DED5	Leipzig	1	DED
72	DE	DEE0	Sachsen-Anhalt	1	DEE
73	DE	DEF0	Schleswig-Holstein	1	DEF
74	DE	DEG0	Thüringen	1	DEG
75	DK	DK01	Hovedstaden	0	DK
76	DK	DK02	Sjælland	0	DK
77	DK	DK03	Syddanmark	0	DK
78	DK	DK04	Midtjylland	0	DK
79	DK	DK05	Nordjylland	0	DK
80	EE	EE00	Eesti	0	EE
81	EL	EL53	Δυτική Μακεδονία	1	EL1
82	EL	EL54	Ήπειρος	1	EL2
83	EL	EL61	Θεσσαλία	1	EL1
84	EL	EL62	Ιόνια Νησιά	1	EL2
85	EL	EL63	Δυτική Ελλάδα	1	EL2

86	EL	EL64	Στερεά Ελλάδα	1	EL2
87	EL	EL65	Πελοπόννησος	1	EL2
88	EL	EL30	Αττική	1	EL3
89	EL	EL41	Βόρειο Αιγαίο	1	EL4
90	EL	EL42	Νότιο Αιγαίο	1	EL4
91	EL	EL43	Κρήτη	1	EL4
92	EL	EL51	Ανατολική Μακεδονία, Θράκη	1	EL1
93	EL	EL52	Κεντρική Μακεδονία	1	EL1
94	ES	ES43	Extremadura	2	ES43
95	ES	ES51	Cataluña	2	ES51
96	ES	ES52	Comunidad Valenciana	2	ES52
97	ES	ES53	Illes Balears	2	ES53
98	ES	ES61	Andalucía	2	ES61-ES63-ES64
99	ES	ES62	Región de Murcia	2	ES62
	ES	ES63	Ciudad Autónoma de Ceuta	2	ES61-ES63-ES64
	ES	ES64	Ciudad Autónoma de Melilla	2	ES61-ES63-ES64
	ES	ES70	Canarias	2	ES70
100	ES	ES11	Galicia	2	ES11
101	ES	ES12	Principado de Asturias	2	ES12
102	ES	ES13	Cantabria	2	ES13
103	ES	ES21	País Vasco	2	ES21
104	ES	ES22	Comunidad Foral de Navarra	2	ES22
105	ES	ES23	La Rioja	2	ES23
106	ES	ES24	Aragón	2	ES24
107	ES	ES30	Comunidad de Madrid	2	ES30
108	ES	ES41	Castilla y León	2	ES41
109	ES	ES42	Castilla-La Mancha	2	ES42
110	FI	FI19	Länsi-Suomi	0	FI
111	FI	FI1B	Helsinki-Uusimaa	0	FI
112	FI	FI1C	Etelä-Suomi	0	FI
113	FI	FI1D	Pohjois- ja Itä-Suomi	0	FI
114	FI	FI20	Åland	0	FI
115	FR	FRI3	Poitou-Charentes	2	FR53
116	FR	FRJ1	Languedoc-Roussillon	2	FR81
117	FR	FRJ2	Midi-Pyrénées	2	FR62
118	FR	FRK1	Auvergne	2	FR72
119	FR	FRK2	Rhône-Alpes	2	FR71
120	FR	FRL0	Provence-Alpes-Côte d’Azur	2	FR82
121	FR	FRM0	Corse	2	FR83
122	FR	FR10	Ile-de-France	2	FR10
123	FR	FRB0	Centre - Val de Loire	2	FR24
124	FR	FRC1	Bourgogne	2	FR26
125	FR	FRC2	Franche-Comté	2	FR43
126	FR	FRD1	Basse-Normandie	2	FR25
127	FR	FRD2	Haute-Normandie	2	FR23

128	FR	FRE1	Nord-Pas de Calais	2	FR30
129	FR	FRE2	Picardie	2	FR22
130	FR	FRF1	Alsace	2	FR42
131	FR	FRF2	Champagne-Ardenne	2	FR21
132	FR	FRF3	Lorraine	2	FR41
133	FR	FRG0	Pays de la Loire	2	FR51
134	FR	FRH0	Bretagne	2	FR52
135	FR	FRI1	Aquitaine	2	FR61
136	FR	FRI2	Limousin	2	FR63
137	HR	HR03	Jadranska Hrvatska	0	HR
138	HR	HR04	Kontinentalna Hrvatska	0	HR
139	HU	HU23	Dél-Dunántúl	0	HU
140	HU	HU31	Észak-Magyarország	0	HU
141	HU	HU32	Észak-Alföld	0	HU
142	HU	HU33	Dél-Alföld	0	HU
143	HU	HU11	Budapest	0	HU
144	HU	HU12	Pest	0	HU
145	HU	HU21	Közép-Dunántúl	0	HU
146	HU	HU22	Nyugat-Dunántúl	0	HU
147	IE	IE04	Northern and Western	0	IE
148	IE	IE05	Southern	0	IE
149	IE	IE06	Eastern and Midland	0	IE
150	IS	IS	Ísland	0	IS
151	IT	ITC1	Piemonte	2	ITC1
152	IT	ITC2	Valle d’Aosta/Vallée d’Aoste	2	ITC2
153	IT	ITC3	Liguria	2	ITC3
154	IT	ITG2	Sardegna	2	ITG2
155	IT	ITH1	Provincia Autonoma di Bolzano/Bozen	2	ITH1-ITH2
156	IT	ITH2	Provincia Autonoma di Trento	2	ITH1-ITH2
157	IT	ITH3	Veneto	2	ITH3
158	IT	ITH4	Friuli-Venezia Giulia	2	ITH4
159	IT	ITH5	Emilia-Romagna	2	ITH5
160	IT	ITI1	Toscana	2	ITI1
161	IT	ITI2	Umbria	2	ITI2
162	IT	ITI3	Marche	2	ITI3
163	IT	ITI4	Lazio	2	ITI4
164	IT	ITC4	Lombardia	2	ITC4
165	IT	ITF1	Abruzzo	2	ITF1
166	IT	ITF2	Molise	2	ITF2
167	IT	ITF3	Campania	2	ITF3
168	IT	ITF4	Puglia	2	ITF4
169	IT	ITF5	Basilicata	2	ITF5
170	IT	ITF6	Calabria	2	ITF6
171	IT	ITG1	Sicilia	2	ITG1
172	LT	LT01	Sostinės regionas	0	LT
173	LT	LT02	Vidurio ir vakarų Lietuvos regionas	0	LT

174	LU	LU00	Luxembourg	0	LU
175	LV	LV00	Latvija	0	LV
176	MT	MT00	Malta	0	MT
177	NL	NL11	Groningen	1	NL1
178	NL	NL12	Friesland (NL)	1	NL1
179	NL	NL13	Drenthe	1	NL1
180	NL	NL21	Overijssel	1	NL2
181	NL	NL22	Gelderland	1	NL2
182	NL	NL23	Flevoland	1	NL2
183	NL	NL31	Utrecht	1	NL3
184	NL	NL32	Noord-Holland	1	NL3
185	NL	NL33	Zuid-Holland	1	NL3
186	NL	NL34	Zeeland	1	NL3
187	NL	NL41	Noord-Brabant	1	NL4
188	NL	NL42	Limburg (NL)	1	NL4
189	NO	NO	Norge	0	NO
190	PL	PL22	Śląskie	1	PL2
191	PL	PL41	Wielkopolskie	1	PL4
192	PL	PL42	Zachodniopomorskie	1	PL4
193	PL	PL43	Lubuskie	1	PL4
194	PL	PL51	Dolnośląskie	1	PL5
195	PL	PL52	Opolskie	1	PL5
196	PL	PL61	Kujawsko-pomorskie	1	PL6
197	PL	PL62	Warmińsko-mazurskie	1	PL6
198	PL	PL63	Pomorskie	1	PL6
199	PL	PL71	Łódzkie	1	PL1
200	PL	PL72	Świętokrzyskie	1	PL3
201	PL	PL81	Lubelskie	1	PL3
202	PL	PL82	Podkarpackie	1	PL3
203	PL	PL84	Podlaskie	1	PL3
204	PL	PL91	Warszawski stołeczny	1	PL1
205	PL	PL92	Mazowiecki regionalny	1	PL1
206	PL	PL21	Małopolskie	1	PL2
207	PT	PT16	Centro (PT)	2	PT16
208	PT	PT17	Área Metropolitana de Lisboa	2	PT17
209	PT	PT18	Alentejo	2	PT18
210	PT	PT11	Norte	2	PT11
211	PT	PT15	Algarve	2	PT15
212	RO	RO11	Nord-Vest	0	RO
213	RO	RO12	Centru	0	RO
214	RO	RO21	Nord-Est	0	RO
215	RO	RO22	Sud-Est	0	RO
216	RO	RO31	Sud - Muntenia	0	RO
217	RO	RO32	București-Ilfov	0	RO
218	RO	RO41	Sud-Vest Oltenia	0	RO
219	RO	RO42	Vest	0	RO
220	SE	SE11	Stockholm	1	SE1

221	SE	SE12	Östra Mellansverige	1	SE1
222	SE	SE21	Småland med öarna	1	SE2
223	SE	SE22	Sydsverige	1	SE2
224	SE	SE23	Västsverige	1	SE2
225	SE	SE31	Norra Mellansverige	1	SE3
226	SE	SE32	Mellersta Norrland	1	SE3
227	SE	SE33	Övre Norrland	1	SE3
228	SI	SI03	Vzhodna Slovenija	0	SI
229	SI	SI04	Zahodna Slovenija	0	SI
230	SK	SK01	Bratislavský kraj	0	SK
231	SK	SK02	Západné Slovensko	0	SK
232	SK	SK03	Stredné Slovensko	0	SK
233	SK	SK04	Východné Slovensko	0	SK
234	UK	UKC1	Tees Valley and Durham	1	UKC
235	UK	UKC2	Northumberland and Tyne and Wear	1	UKC
236	UK	UKD1	Cumbria	1	UKD
237	UK	UKD3	Greater Manchester	1	UKD
238	UK	UKD4	Lancashire	1	UKD
239	UK	UKD6	Cheshire	1	UKD
240	UK	UKD7	Merseyside	1	UKD
241	UK	UKE1	East Yorkshire and Northern Lincolnshire	1	UKE
242	UK	UKE2	North Yorkshire	1	UKE
243	UK	UKE3	South Yorkshire	1	UKE
244	UK	UKM6	Highlands and Islands	1	UKM
245	UK	UKK1	Gloucestershire, Wiltshire and Bristol/Bath area	1	UKK
246	UK	UKK2	Dorset and Somerset	1	UKK
247	UK	UKK3	Cornwall and Isles of Scilly	1	UKK
248	UK	UKK4	Devon	1	UKK
249	UK	UKL1	West Wales and The Valleys	1	UKL
250	UK	UKL2	East Wales	1	UKL
251	UK	UKM5	North Eastern Scotland	1	UKM
252	UK	UKM7	Eastern Scotland	1	UKM
253	UK	UKM8	West Central Scotland	1	UKM
254	UK	UKM9	Southern Scotland	1	UKM
255	UK	UKN0	Northern Ireland	1	UKN
256	UK	UKE4	West Yorkshire	1	UKE
257	UK	UKF1	Derbyshire and Nottinghamshire	1	UKF
258	UK	UKF2	Leicestershire, Rutland and Northamptonshire	1	UKF
259	UK	UKF3	Lincolnshire	1	UKF
260	UK	UKG1	Herefordshire, Worcestershire and Warwickshire	1	UKG
261	UK	UKG2	Shropshire and Staffordshire	1	UKG
262	UK	UKG3	West Midlands	1	UKG
263	UK	UKH1	East Anglia	1	UKH
264	UK	UKH2	Bedfordshire and Hertfordshire	1	UKH
265	UK	UKH3	Essex	1	UKH
266	UK	UKI3	Inner London - West	1	UKI
267	UK	UKI4	Inner London - East	1	UKI

268	UK	UKI5	Outer London - East and North East	1	UKI
269	UK	UKI6	Outer London - South	1	UKI
270	UK	UKI7	Outer London - West and North West	1	UKI
271	UK	UKJ1	Berkshire, Buckinghamshire and Oxfordshire	1	UKJ
272	UK	UKJ2	Surrey, East and West Sussex	1	UKJ
273	UK	UKJ3	Hampshire and Isle of Wight	1	UKJ
274	UK	UKJ4	Kent	1	UKJ

Supplementary Table 3. Classification of economic sectors. Five macro-sectors were distinguished attending to their heat exposure levels and working load intensity in Watts (W).

Economic Sector	Abbreviation	Heat Exposure	Metabolic Intensity (W)
Agriculture	AGR	Outdoor	High
Industry	IND	Indoor	Medium
Construction	CONST	Outdoor	High
Transportation	TRANS	Outdoor	Medium
Services	SERV	Indoor	Low

Supplementary Discussion

The physical impacts of climate change considered in our study (based on the RCP8.5 scenario) are only compatible with the socioeconomic scenario SSP5 (as illustrated by Fig. SD1). This limits the possibilities of considering different RCP-SSP scenarios to a single combination: RCP8.5-SSP5. We replicated in our model the GDP and population patterns implied by SSP5, as obtained from the IIASA SSP database (https://tntcat.iiasa.ac.at/SspDb/dsd?Action=htmlpage&page=10), considering that the stock of labour follows the same growth pattern shown by the population and that subnational regions follow the projections of the country they belong to. We imposed exogenously the population and labour dynamics in the CGE and calibrated the GDP using the Total Factor Productivity (TFP) to meet the SSP5 targets. Meanwhile, capital accumulation takes place endogenously in the CGE via the recursive addition of investment coming from the previous period.

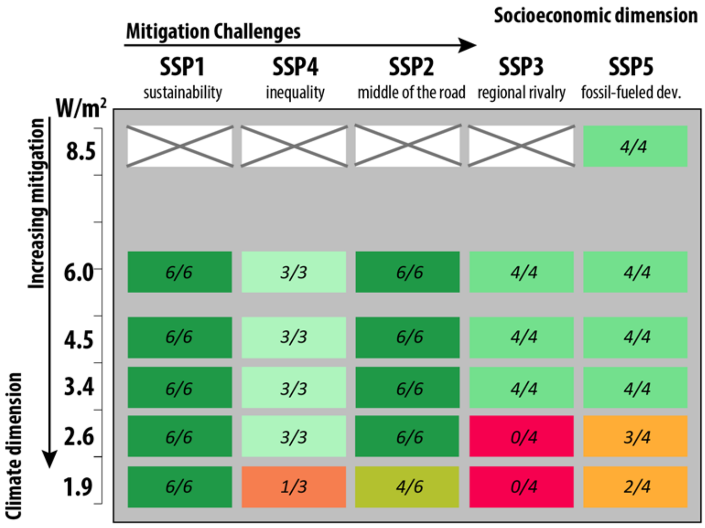

Figure SD1. (from Rogelj et al. 2018, Supplementary Information) Overview of available scenario runs in the SSP-RCP matrix framework. Values in each box represent the number of available scenario runs over the number of participating modelling frameworks. Given that used climate data forced by RCP8.5, the only SSP scenario compatible with this data is SSP5.

The results of this additional experiment offer interesting insights. We observe that, in general, economic losses tend to be lower in Europe (Fig. SD2) compared to the previous set of results (Fig. 4a) based on a comparative static framework.

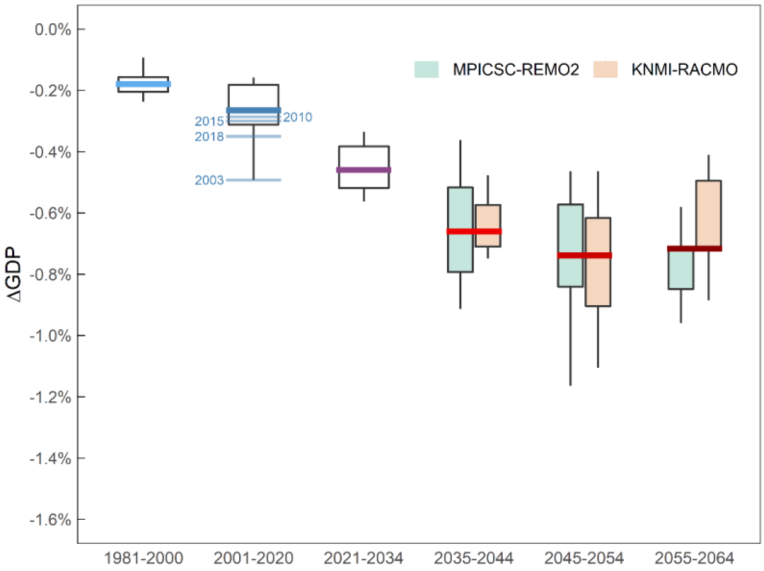

Figure SD2. Evolution of economic damages in Europe considering a dynamic framework RCP8.5-SSP5 characterised by a push for economic and social development coupled with the exploitation of abundant fossil fuel resources and absence of climate policies. Boxes cover the interquartile range (IQR, 25th-75th percentiles) of the damage distribution and whiskers show the values contained within ±1.5·IQR.

The main driver for this difference is the fact that capital becomes more important in the structure of the future economy. Since the productivity impacts of heatwaves only affect labour, this has a direct consequence on the final allocation of factors. In this setup, the exogenous growth in population (labour) is not sufficient to guarantee a high macroeconomic growth path, as imposed by the SSP5. Hence, capital grows more relative to labour and accumulates in the economy. Thus, the decrease in total damages in response to labour shocks. This higher adaptive capacity shown under the combination RCP8.5-SSP5 can also be observed, for example, in Orlov et al. (2020).

Changes in the demographic composition across regions play a limited role, as population is expected to grow strongly in Northern Europe, grow moderately in Mediterranean Europe and remain stable or even declining in Eastern Europe. Since the hardest impacts of heatwaves are concentrated in Southern Europe, this variable does not influence dramatically the final impact. In contrast, one key driver appears to be the sectoral structure of the economy, especially the weight of outdoor and indoor economic activities projected for the future. While in the comparative-statics experiment, the sectoral composition remains fixed, in the dynamic setup the economy develops according to the GDP and population targets, which are exogenous in the SSP5 but the sectoral composition responds to different market mechanisms, which are all endogenous and confounded in the CGE. These market mechanisms refer to trade specialisation (based on regional comparative advantages), primary factor reallocation across sectors, investment dynamics and to how all these forces interact with the GDP and population targets. This makes the economic structure of some sub-national regions to be changed substantially over time leading to, for example, a retreat of impacts in some southern European economies in the last decade of our analysis (Fig. SD3).

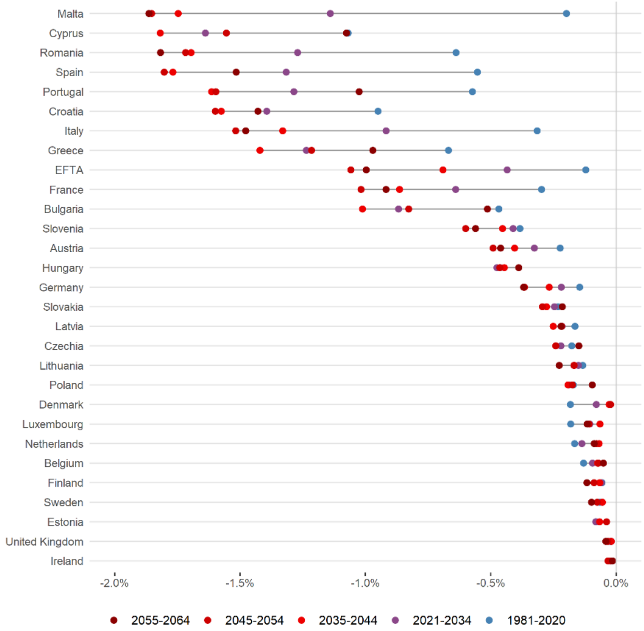

Figure SD3. Evolution of heatwave-derived economic damages at the country level under the RCP8.5-SSP5 dynamic scenario.

One way to restrict severe sector reallocations would be to calibrate the sectoral economic composition of the NUTS-2 regions, but this could be computationally infeasible and would also imply a certain degree of arbitrariness, as SSPs do not provide information about the sectoral evolution.

These results point us to existing endogenous mechanisms through which the European economy would partly absorb the projected increasing heat load of work. However, results should be interpreted cautiously, as they are based on a specific future scenario (SSP5) featuring strong economic growth and technological progress as well as rapid and costless adaptation. Some other aspects should also be controlled with more detail as, for example, the evolution of the sectoral economic composition. In addition, because assumptions of demographic and economic developments over long-time spans are highly uncertain (Dellink et al., 2017; Christensen et al., 2018), it would be desirable to perform a comprehensive assessment covering the whole spectrum of RCP-SSP scenarios to account for these uncertainties.

Supplementary References

Christensen P, Gillingham K, Nordhaus W (2018) Uncertainty in forecasts of long-run economic growth. Proc. Natl Acad. Sci. USA 115, 5409–5414.

Dellink R, Chateau J, Lanzi E, Magné, B (2017) Long-term economic growth projections in the shared socioeconomic pathways. Glob. Environ. Change 42, 200–214.

Orlov A, Sillman J, Aunan K, Kjellstrom T, Aaheim A (2020) Economic costs of heat-induced reductions in worker productivity due to global warming. Global Environmental Change 63:102087.

Rogelj J, Popp A, Calvin KV, et al (2018) Scenarios towards limiting global mean temperature increase below 1.5 °C. Nature Clim Change 8, 325–332.
