# Supplementary material for: Current and projected regional economic impacts of heatwaves in Europe
Source: Nat Commun. 2021 Oct 4;12:5807. doi: 10.1038/s41467-021-26050-z (PMC8490455; doi:10.1038/s41467-021-26050-z)
Supplement: Supplementary file 3 — Reporting Summary [file 41467_2021_26050_MOESM3_ESM.pdf]

nature portfolio

Corresponding author(s): David García-León

Last updated by author(s): Aug 29, 2021

Reporting Summary

Nature Portfolio wishes to improve the reproducibility of the work that we publish. This form provides structure for consistency and transparency in reporting. For further information on Nature Portfolio policies, see our Editorial Policies and the Editorial Policy Checklist.

Statistics

For all statistical analyses, confirm that the following items are present in the figure legend, table legend, main text, or Methods section.

n/a	Confirmed	
☒	☐	The exact sample size (n) for each experimental group/condition, given as a discrete number and unit of measurement
☒	☐	A statement on whether measurements were taken from distinct samples or whether the same sample was measured repeatedly
☒	☐	The statistical test(s) used AND whether they are one- or two-sided<br>*Only common tests should be described solely by name; describe more complex techniques in the Methods section.*
☒	☐	A description of all covariates tested
☒	☐	A description of any assumptions or corrections, such as tests of normality and adjustment for multiple comparisons
☒	☐	A full description of the statistical parameters including central tendency (e.g. means) or other basic estimates (e.g. regression coefficient) AND variation (e.g. standard deviation) or associated estimates of uncertainty (e.g. confidence intervals)
☒	☐	For null hypothesis testing, the test statistic (e.g. F, t, r) with confidence intervals, effect sizes, degrees of freedom and P value noted<br>*Give P values as exact values whenever suitable.*
☒	☐	For Bayesian analysis, information on the choice of priors and Markov chain Monte Carlo settings
☒	☐	For hierarchical and complex designs, identification of the appropriate level for tests and full reporting of outcomes
☒	☐	Estimates of effect sizes (e.g. Cohen's d, Pearson's r), indicating how they were calculated

Our web collection on statistics for biologists contains articles on many of the points above.

Software and code

Policy information about availability of computer code

Data collection	ERA5-Land data were collected using the Copernicus' Climate Data Store Application Program Interface (CDS API) (https://cds.climate.copernicus.eu/api-how-to).
Data analysis	R (v. 3.6.0) was used to implement the two versions of the WBGT used in this study using the package HeatStress (https://github.com/anacv/HeatStress - https://doi.org/10.5281/zenodo.3264929), under license GPL-3. Regional heatwave-induced productivity shocks were also calculated with R and assembled in MS Excel. The economic model was simulated using GEMPACK (v. 12.0) and the results of the simulations were analysed in R. Maps shown in Figs. 1,2 were rendered in QGIS (v. 3.16.1). The remaining graphical outputs were obtained in R using the package ggplot2 (v. 3.3.2).

For manuscripts utilizing custom algorithms or software that are central to the research but not yet described in published literature, software must be made available to editors and reviewers. We strongly encourage code deposition in a community repository (e.g. GitHub). See the Nature Portfolio guidelines for submitting code & software for further information.

Data

Policy information about availability of data

All manuscripts must include a data availability statement. This statement should provide the following information, where applicable:

- Accession codes, unique identifiers, or web links for publicly available datasets
- A description of any restrictions on data availability
- For clinical datasets or third party data, please ensure that the statement adheres to our policy

The historical climate data that support the findings of this study are publicly available at the Copernicus Climate Data Store (https://doi.org/10.24381/cds.bd0915c6). Future climate projections are available for download via the Earth System Grid Federation (ESGF, https://esgf.llnl.gov/) under the project name

"CORDEX" at any of the ESGF nodes, such as for example, https://esgf-node.ipsl.upmc.fr/search/cordex-ipsl/. The Social Accounting Matrices (GTAP) used to calibrate the economic model were used under license for the current study, and so are not publicly available. Data are however available from the authors upon reasonable request and with permission of the Center for Global Trade Analysis, Department of Agricultural Economics, Purdue University. UN WPP-Adjusted Population Count, v4.11 are available for download at https://sedac.ciesin.columbia.edu/data/set/gpw-v4-population-count-adjusted-to-2015-unwpp-country-totals-rev11. Quarterly sectoral accounts used in the seasonal adjustment of productivity shocks were obtained from Eurostat (https://ec.europa.eu/eurostat/databrowser/view/namq_10_gdp/default/table?lang=en).

Field-specific reporting

Please select the one below that is the best fit for your research. If you are not sure, read the appropriate sections before making your selection.

- [ ] Life sciences
- [x] Behavioural & social sciences
- [ ] Ecological, evolutionary & environmental sciences

For a reference copy of the document with all sections, see nature.com/documents/nr-reporting-summary-flat.pdf

Behavioural & social sciences study design

All studies must disclose on these points even when the disclosure is negative.

Study description	We quantitatively estimate the costs associated with excessive heat at the workplace in Europe by integrating WBGT-derived productivity losses into a regionalised computable general equilibrium model. We apply this methodology to heatwaves occurring in four recent anomalously hot years and to short- and mid-future heat scenarios stemming from climate model projections.
Research sample	Not applicable. We studied all the available regions in Europe (EU-27 + UK + EFTA countries). We studied all years in the period 1981-2020, with a focus on 2003, 2010, 2015, and 2018, as these years are examples of anomalously hot years. For future projections, we focused on two climate models spanning a wide range of future climate conditions (more details on the choice of climate models are provided in the main text and the methods).
Sampling strategy	No sampling strategy was required. We studied all the events and regions available.
Data collection	Climate data (ERA5 hourly data) were retrieved from Copernicus (European Commission's Climate Change Service). Climate projections were retrieved from EURO-CORDEX. Population data and population projections were downloaded from CIESIN (Columbia University). The economic model was calibrated using the GTAP database (version 8) and was spatially disaggregated using regional economic data from EUROSTAT.
Timing	Spatial scale: Europe; Temporal scope: 1981-2064
Data exclusions	No data were excluded from the analyses
Non-participation	This field is not applicable to the study. All regions/years/events were considered in our analysis.
Randomization	This field is not applicable to the study. All regions/years/events were considered in our analysis.

Reporting for specific materials, systems and methods

We require information from authors about some types of materials, experimental systems and methods used in many studies. Here, indicate whether each material, system or method listed is relevant to your study. If you are not sure if a list item applies to your research, read the appropriate section before selecting a response.

Materials & experimental systems

n/a	Involved in the study	
x		Antibodies
x		Eukaryotic cell lines
x		Palaeontology and archaeology
x		Animals and other organisms
x		Human research participants
x		Clinical data
x		Dual use research of concern

Methods

n/a	Involved in the study	
x		ChIP-seq
x		Flow cytometry
x		MRI-based neuroimaging
